# Physical Therapy Programs in Older Adults with Coronary Artery Disease: Preferences to Technology-Based Cardiac Physical Therapy Programs

**DOI:** 10.3390/ijerph192013130

**Published:** 2022-10-12

**Authors:** Elena Marques-Sule, Nuria Sempere-Rubio, Sergio Esparcia-Sánchez, Pallav Deka, Trinidad Sentandreu-Mañó, Juan Luis Sánchez-González, Leonie Klompstra, Noemí Moreno-Segura

**Affiliations:** 1 Physiotherapy in Motion, Multispeciality Research Group (PTinMOTION), Department of Physiotherapy, University of Valencia, Gasco Oliag 5, 46010 Valencia, Spain; 2 Clinical Biomechanics Research Unit (UBIC), Department of Physiotherapy, Universitat de València, Gasco Oliag 5, 46010 Valencia, Spain; 3 Department of Physiotherapy, University of Valencia, Gasco Oliag 5, 46010 Valencia, Spain; 4 College of Nursing, Michigan State University, East Lansing, MI 48824, USA; 5 Department of Nursing and Physiotherapy, University of Salamanca, 37007 Salamanca, Spain; 6 Department of Health, Medicine and Caring Sciences, Linkoping University, 58185 Linkoping, Sweden

**Keywords:** physical therapy, coronary artery disease, technology-based physical therapy, exercise, healthy lifestyle, ischemic heart disease, older adults

## Abstract

(1) Background: Assessing preferences in technology-based cardiac physical therapy programs in older adults with coronary artery disease (CAD) is fundamental to promoting adherence to healthy lifestyles and healthy aging. This study aimed at analyzing preferences in technology-based cardiac physical therapy programs in older adults with CAD. Additionally, a comparison by sex was performed. (2) Methods: Cross-sectional study. 70 older adults with CAD (mean age 66.73 ± 0.77, 80% men) were evaluated. Technology use and preferences in technology-based cardiac physical therapy programs (Technology Usage Questionnaire) were assessed. (3) Results: 97.1% of the sample had Smartphones and 81.4% accessed the Internet every day, mostly with their Smartphones (75.5%). A total of 54.3% were interested in receiving rehabilitation via their Smartphone, and most of the sample considered ideas to manage stress (92.9%), healthy meal ideas and recipes (85.7%), exercise ideas (84.3%), exercise prompts (72.9%), setting goals (67.1%), exercise taught by a virtual therapist (65.7%), ideas to overcome cigarette cravings (62.9%), information on local exercise opportunities (60%), ideas to remember to take medications (57.1%), steps to achieve goals (54.3%) and eating tips for takeaways (51.7%) very useful. Additionally, men considered the technology-based advice about exercise prompts, healthy meal ideas and recipes, and ideas to manage stress more useful than women, and had more frequently a Smartphone, less frequently made phone calls, had more regular access to the Internet, and used the Internet more often. (4) Conclusions: Clinicians should encourage older adults to engage in cardiac technology-based physical therapy programs to provide meaningful exercise counselling, promote healthy lifestyle and healthy aging.

## 1. Introduction

One of the most important events of the 21st century is that the world population is ageing and, although this is somewhat desirable, the fact is that the number of people over 60 years of age is increasing more than other groups. Therefore, a population with comorbidities is growing at the same time [1,2]. Thus, it represents a global challenge that requires effective, feasible and resource-adjusted strategies to address this problem in order to achieve healthy ageing, that is, positive ageing, free of diseases and with functional capacity maintained, which allows well-being in older adults. Regarding this concept, aging in a healthy way means being able to do things that people value as long as possible [3].

Coronary artery disease (CAD) is defined as the pathological process characterized by the accumulation of atherosclerotic plaque in the coronary arteries that triggers arterial narrowing and reduces heart flow (obstructive or not), and is a common issue in older adults in the developed world which has considerable impact on their morbimortality [4].

Cardiac physical therapy programs (CPP), which focus on physical and psychosocial recovery and on the promotion of healthy lifestyles, have a pivotal role in the standard treatment. CPP aims at reducing the progression of CAD and at obtaining an improvement in older adults’ health. It focuses on the underlying cause of cardiovascular disease and thus promotes healthy aging; so, older adults can preserve and resume optimal functioning in their community [5,6,7]. However, long-term adherence to CPP programs is extremely low [7,8].

Tele-rehabilitation (TR) is a new alternative that has revolutionized physical therapy programs, and can be defined as a rehabilitation treatment based on the use of information and communication technologies [9]. Published evidence suggests that clinical results of TR are equal to or better than in-person rehabilitation programs. Moreover, TR programs improve self-management skills such as self-efficacy and independent exercise planification, obtaining long-term improvements in physical activity, quality of life and reduction of cardiovascular risk factors [10,11,12]. In general, TR programs are better adapted to patient preferences (time and location flexibility), thereby achieving greater satisfaction and adherence [8,10,13,14]. Furthermore, the use of portable sensors integrated into smartwatches and Smartphones may play a key role in providing information to physical therapists related to physical activity performance, and also allowing therapists to adjust the parameters of intensity, frequency, and levels of physical activity. Therefore, taking into account all of these reasons, as well as the fact of the increased use of mobile phones in older adults, TR could be a useful alternative to in-person CPP, in order to improve the rates of adherence, satisfaction, healthy lifestyle and healthy aging [15].

However, although in the general population the usability, usefulness, acceptability and acceptance of cardiac TR seems to be high, there may be other factors that influence the acceptability of these programs, such as the patient’s knowledge about technologies, taking into account that an efficient use would imply a greater use of TR [16]. Consequently, the use of simple technology-based programs, as well as implementing digital literacy programs for older adults, may be essential to achieve favorable results and address the needs of older patients with CAD [16,17]. Further, assessing preferences in technology-based cardiac physical therapy programs in older adults with CAD is fundamental to improving home-based protocols, adherence to healthy lifestyles and to promote healthy aging.

The main objective of this study was to analyze the preferences in technology-based CPP in older adults with CAD. Additionally, a comparison by sex was performed.

## 2. Materials and Methods

### 2.1. Study Design

A cross-sectional study was undertaken in the present study at the University of Valencia, Valencia (Spain). Participants were recruited from cardiac rehabilitation centers.

### 2.2. Participants

All participants were informed about the purpose and content of the project and provided written informed consent to participate in the study. All procedures were approved by the Committee of Ethics in Experimental Research of the University of Valencia (IE1529270) and complied with the requirements listed in the 1975 Declaration of Helsinki and its amendment in 2008.

### 2.3. Sample

A total sample of 70 subjects was included in this study. Inclusion criteria were having a diagnosis of CAD and being able to maintain independent ambulation. Those who presented recent symptoms of chest pain, decompensated hypertension or any other musculoskeletal condition that could affect the ability to perform physical activity were excluded.

### 2.4. Measurements

First, an interview with the study participants was conducted to collect sociodemographic information (age, sex, marital status, and employment status).

The technology usage of older adults was assessed with the Technology Usage Questionnaire [15], concretely with 10 items of the questionnaire that evaluate the following aspects: (a) Ownership and the use of mobile devices and/or Smartphones; (b) Availability and access to the Internet; (c) Type of devices used in order to access to the Internet; (d) Use of physical activity games (for example, the Nintendo Wii); (e) Use of heart rate monitors and other devices to monitor the physical activity (such as pedometers, Fitbit, etc.).

Second, preferences of patients regarding technology-based CPP were assessed with the Technology Usage Questionnaire [15], concretely with 12 questions that include the basic elements of CPP (physical activity, stress management and tobacco cessation). In this questionnaire, the subject is instructed to answer as to how useful is the advice of these elements for them (scores ranging from 0 to 5, with 0 not being useful and 5 being very useful) and other questions related to the time spent in sport activities, as well as their motivation to perform them. The main aspects evaluated are: (a) Interest in carrying out a technology-focused CPP via the Internet; (b) CPP support through e-mail, educational videos, web sites or leaflets; (c) Interests in the possibility of carrying out CPP at home through computer games or virtual healthcare; (d) Preferred form of interaction with the online platform; (e) Benefits of health advice on the different elements of the virtual CPP session; (f) Practical usefulness of the advice given on the exercise prompts, exercise ideas, exercise program taught by a virtual therapist, information on local exercise opportunities, setting goals, steps to achieve goals, how to overcome cigarette cravings, how to remember to take your medications, healthy meal ideas and recipes, healthy eating tips for takeaways, utility of practical ideas to manage stress and dining out or how to link up with others who are living with heart disease; (g) Interest in receiving monitoring of parameters and online feedback; and (h) Opinion and interest in CPP treatment based on technology in general.

### 2.5. Statistics

Analysis of survey responses was largely descriptive. Items were re-coded such that 1 equaled a positive response (i.e., “yes”), while 0 indicated a negative response. Gender was coded as one for male and two for female. Descriptive measurements were shown as mean, with their standard deviation, or frequencies. Moreover, the Chi square test was employed to find differences between sex in qualitative variables. Statistical significance was set for all statistical tests with a degree of significance *p* < 0.05. All statistical analyses were carried out in IBM SPSS Statistics software (Version 22.0, IBM Corp, Armonk, NY, USA).

## 3. Results

### 3.1. Sociodemographic and Characterisctics of the Sample

A total of 70 patients constituted the final sample. Sociodemographic variables are depicted in Table 1. A total of 80% of participants were male [mean age 66.57 ± 5.83 (range 57–80) years] and 20% were female [mean age 67.35 ± 5.66 (range 59–79) years]. Regarding sociodemographic characteristics, most of the sample had secondary education, were retired, did not present comorbidities, practiced exercise more than 3 times per week, did not smoke or consume alcohol, and their main reason to attend cardiac rehabilitation was having suffered a myocardial infarction. No differences were found by sex in the following variables: comorbidities, frequency of physical exercise, smoking, alcohol consumption and reason for cardiac rehabilitation (*p* > 0.05), although statistically significant differences were found with respect to education (χ^2^ = 13.2, *p* = 0.021) and employment (χ^2^ = 23.4, *p* = 0.001).

### 3.2. Comparison of Technology Usage of Older Adults by Sex

With regard to technology usage, most of the sample had a mobile phone, and used the following functions: phone calls, text messages, camera, receiving photos and videos, Internet search, applications, instant messaging, social networks and gaming applications. Moreover, most of the sample used a Smartphone to access the Internet every day. The most used devices to access the Internet were personal computers and Smartphones. When comparing by sex, there was a significant association between patients who had or do not have a mobile phone or Smartphone, and sex (χ^2^ = 8.235, *p* = 0.04), with a low to moderate relationship. Also, the use of a mobile phone for phone calls (χ^2^ = 4.0, *p* = 0.044), regular access to the Internet (χ^2^ = 6.8, *p* = 0.009) and frequency of Internet use (χ^2^ = 9.1, *p* = 0.027) presented a low-to-moderate association when comparing by sex. No further significant associations were found for the rest of the variables analyzed by sex (*p* > 0.05) (Table 2).

Heart rate monitors to measure heart rate during exercise sessions were used by 60% of the participants (50% of women and 62.50% of men).

### 3.3. Preferences of Older Adults towards Technology-Based CPP Programs

Regarding preferences in technology-based CPP programs, most of the participants were interested in receiving continuing advice and support about cardiac rehabilitation through a mobile phone/Smartphone, and the most preferred form of communication was email. Most of the participants would like to sign up to a free program that offered this type of communication, although they would prefer not receiving messages. No significant association was found in any of the variables when comparing by sex (*p* > 0.05) (Table 3). In addition, all patients were asked whether they were interested in receiving continuing CPP support via the Internet, as well as about the preferred Internet communication function (e-mail, educational video, web sites or leaf form). The χ^2^ test showed no significant association in any variable with respect to sex (*p* > 0.05). Finally, all patients were asked whether they thought it would be useful to perform a CPP through Nintendo Wii, as well as the level of interaction (38% of patients preferred an interaction based on a few mouse clicks, and 24% preferred a full menu to interact with the platform), as well as whether they considered virtual rehabilitation useful. When comparing by sex, the χ^2^ test showed no significant associations in any variable (*p* > 0.05).

Regarding preferences of older adults regarding advice related to technology-based cardiac physical therapy programs, most of the participants considered exercise ideas, exercise prompts, exercise program taught by a virtual therapist, information on local exercise opportunities, healthy meal ideas and recipes, practical ideas to manage stress, setting goals, steps to achieve goals, ideas to overcome cigarette cravings, ideas to remember how to take medication and healthy eating tips for takeaways and dining out very useful. By contrast, the majority of the participants considered that linking up with others who are living with heart disease through the program was not useful. With respect to the usefulness of exercise prompts in technology-based CPP, there was a statistically significant association between exercise indications and sex (χ^2^ = 14.73, *p* = 0.005), with a moderate relationship. Similar results were found regarding the association between the utility of healthy meal ideas and recipes (χ^2^ = 8.35, *p* = 0.015) and the utility of practical ideas to manage stress (χ^2^ = 6.62, *p* = 0.036) and sex, with a low to moderate relationship. No further significant relationships were found for the rest of the variables analyzed by sex (exercise ideas, exercise program taught by a virtual coach, information on local exercise opportunities, setting goals, steps to achieve goals, how to overcome cigarette cravings, how to remember to take your medications, healthy eating tips for takeaways and dining out or how to link up with others who are living with heart disease) (*p* > 0.05) (Table 4).

A total of 94.6% of men and 50% of women reported they were not interested in wearing any other type of device to measure their physical activity level.

## 4. Discussion

This is the first study that shows the technology use and preferences in technology-based cardiac physical therapy programs of older adults with CAD. Our study suggests that patients with CAD are interested in technology-based cardiac physical therapy but do not present many differences by sex in the variables that analyze the use of technology, as well as preferences in technology-based physical therapy programs. In this regard, differences were found in having a Smartphone, use of the Smartphone, regular access to the Internet, and frequency of Internet use. By contrast, older men made phone calls less frequently. Moreover, older men found exercise, healthy meal, recipe and managing stress ideas more helpful than older women with CAD.

This information is relevant given the fact that technological progress in mobile applications, social networks, wearable sensors for vital signs and virtual games is more immediate every day. Then, an approach using new technologies to the elderly seems to be a challenge that needs to be overcome in order to promote healthy aging [18]. In addition, a lot of studies show that home exercise based interventions can be as effective as center-based programs [19,20,21]. Our results are in the line with previous studies. Dale et al., (2014) [22] reported that 95% of participants had a mobile phone, whilst Buys et al., (2016) [15] reported that 97% of participants had a mobile phone, and 64% of them had a Smartphone. The present study shows a high rate of participants with a mobile phone, concretely 100% of men and 85.7% of women. The evidence suggests that, currently, technology use seems to be high in a large percentage of the population and thus could be employed to progressively introduce cardiac telerehabilitation programs, as well as to increase adherence and even patient satisfaction [14].

Regarding access to the Internet, we found significant differences between men (87.5%) and women (57.1%). By contrast, Peels et al., (2013) [23], Or et al., (2009) [24] and Baron et al., (2012) [25] did not find differences by sex in this regard. On the other hand, our study shows a low percentage of participants used physical activity games such as those available on the Nintendo Wii, both for older men (10.8%) and for women (8.6%). These results are similar to another study (2015) [26] where only 3% of the participants used such physical activity games. By contrast, a higher percentage of subjects (22%) reported having experience with this type of game in another study [15]. Moreover, 62.5% of men and 35.5% of women used mobile applications, contrarily to the study of Pouchieu et al., (2015) [27], who reported that middle-aged women showed a low interest in applications.

On the other hand, both physical activity and the decrease of sedentary behaviors could reduce several comorbidities. Therefore, the use of technologies and devices focused on increasing physical activity could improve wellbeing [28,29]. In our study, half of the participants (44.6% of men and 50% of women) were not interested in receiving continuing advice about cardiac rehabilitation in their mobile phone or Smartphone, primarily because of concerns about their data privacy after registering in the virtual applications or games. Other concerns of the sample were the uncertainty about the benefits of this type of physical therapy and the complexity of use when using technologies. These results are in line with the review performed by Peek et al., (2014) [30], who observed that some of the main reasons for the low use of technologies are the fear of ineffectiveness due to the complexity of use, lack of privacy and cost. This could be a reason, along with the fact that a significant proportion of our sample consisted of older adults, that explains the low interest in a game-based physical therapy program.

A total of 62.5% of men and 50% of women in our sample used heart rate monitors while performing exercise. However, it should be noted that in our sample, 96.60% of men and 85.70% of women who did not use heart rate monitors were not interested in using another type of monitoring device. By contrast, Buys et al., (2016) [15] pointed out that 68% of the patients who did not use heart rate monitors during exercise considered monitoring their heart rate as important, specifically to determine the intensity of home-based programs of exercise.

Lastly, regarding the preferences of older adults about the useful advice of technology-based cardiac physical therapy programs, several outcomes such as exercise ideas, exercise prompts, healthy meal ideas and recipes, practical ideas to manage stress and how to overcome cigarette cravings were considered very useful by the sample. These results are similar to those obtained by Buys et al. [15], who observed that information on exercise ideas, exercise prompts, local exercise opportunities, healthy meal and recipes and suggestions to manage stress were motivators to sign up in a technologic platform focused in CR. Additionally, we found that women were more likely than men to enroll in a CR program with the underlying motivation of socializing and meeting other patients with CAD. Such findings have also been reported by other studies. As such, interventions to promote CR have to be gender specific if desired outcomes for adherence to CR goals are to be achieved. The fact of meeting other people with CAD was not useful for 69.60% of men and 50% of women, as observed in another study [15]. However, it should be taken into account that the present study has some limitations. Firstly, a small sample size consisting of a larger proportion of males (80%) than females (20%) was included. This difference in the participants’ sex may affect the outcomes of the study if an equal number of participants from both sexes were included. In this sense, it should be taken into account that methodological bias could influence the results of this study. However, it should be noted that the percentages reported in this study are similar to attendance rates to cardiac physical therapy programs in Spain, thus it could be the underlying cause of this difference in participants’ sex. Nevertheless, further studies should be performed with larger sample sizes as well as considering a similar distribution of males and females. Secondly, the study was carried out in a single center, therefore results may not be generalizable to the entire population with CAD.

In summary, the future of technology-based physical therapy programs seems to be promising, as an increasing percentage of the population uses new technologies. In addition, due to the health situation experienced during the COVID-19 pandemic, the percentage of face-to-face physical therapy programs has been reduced, whilst uptake and use of remote technologies has increased allowing services to be delivered to patients’ home. Finally, to promote healthy aging, future research should investigate the usefulness of technology-based CR programs in promoting adherence to CR guidelines, to test its impact on improving physical activity, functional capacity and overall quality of life in patients with CAD.

## 5. Conclusions

In conclusion, most older adults with CAD had Smartphones and accessed the Internet every day. In addition, most of them were interested in receiving rehabilitation via their Smartphone, and considered ideas to manage stress, healthy meal ideas and recipes, exercise ideas, exercise prompts, setting goals, exercise taught by a virtual therapist, ideas to overcome cigarette cravings, information on local exercise opportunities, ideas to remember to take medications, steps to achieve goals, and eating tips for takeaways in technology-based cardiac physical therapy programs very useful. Additionally, older men considered technology-based exercise prompts, information on healthy meals and recipes, and ideas to manage stress more useful than older women, and had greater use of Smartphones, made phone calls less frequently, accessed the Internet more regularly, and used the Internet more frequently. The overall use of technology has improved over the years, however, some gender-specific considerations need to be made to motivate greater uptake of technology-based cardiac rehabilitation programs and adherence to cardiac rehabilitation guidelines.

## Figures and Tables

**Table 1:** Sociodemographic characteristics of the participants.

Variable	Total		Male (n = 56; 80%)		Female (n = 14; 20%)		χ^2^ (*p*-Value)
	N	Total%	N	Total%	N	Total%	
Comorbidities							3.2 (0.663)
No comorbidities	27	38.6	22	39.3	5	35.7	
Hypertension	17	24.3	12	21.4	5	35.7	
Diabetes	18	25.7	16	28.6	2	14.3	
Thrombosis	1	1.4	1	1.8	0	0	
Peripheric artery disease	5	7.1	1	1.8	1	7.1	
Other comorbidities	2	2.9	4	7.1	1	7.1	
Education							13.2 (0.021) *
No studies	3	4.3	1	1.8	2	14.3	
Uncompleted primary studies	8	11.4	6	10.7	2	14.3	
Primary	15	21.4	9	16.1	6	42.9	
Secondary	23	32.9	22	39.3	1	7.1	
University	7	10	5	8.9	2	14.3	
Graduate	14	20	13	23.2	1	7.1	
Employment							23.4 (0.001) *
Employed	21	30	19	33.9	2	14.3	
Self-employed	5	7.1	5	8.9	0	0	
Unemployed/looking for	4	5.7	4	7.1	0	0	
Unemployed/without looking for	3	4.3	3	5.4	0	0	
Housewife	6	8.6	1	1.8	5	35.7	
Student	1	1.4	0	0	1	7.1	
Retired	30	42.9	24	42.9	6	42.9	
Frequency of physical exercise							3.5 (0.465)
Less than 1 time/week	8	11.4	6	10.7	2	14.3	
1 time/week	3	4.3	3	5.4	0	0	
2 times/week	1	1.4	1	1.8	0	0	
3 times/week	7	10	4	7.1	3	21.4	
>3 times/week	51	72.9	42	40.8	9	64.3	
Smoking							0.4 (0.508)
Yes	20	28.6	17	30.4	3	21.4	
No	50	71.4	39	69.6	11	78.6	
Alcohol consumption							0.2 (0.634)
Yes	12	17.1	9	16.1	3	21.4	
No	58	82.9	47	83.9	11	78.6	
Reason for cardiac rehabilitation							6.9 (0.14)
Myocardial infarction	16	22.9	14	25	2	14.3	
Stent	7	10	7	12.5	0	0	
Myocardial infarction and stent	43	61.4	33	58.9	10	71.4	
Valve surgery	3	4.3	2	3.6	1	7.1	
Heart failure	1	1.4	0	0	1	7.1	

* Indicates statistically significant differences (*p* < 0.05).

**Table 2:** Comparison of technology usage of older adults by sex.

Variable	Total N (%)	Male N (%)	Female N (%)	χ^2^ (*p*-Value)
1—Have a mobile phone or Smartphone				8.2 (0.04) *
Yes	68 (97.1)	56 (100)	14 (85.7)	
No	2 (2.9)	0 (0)	2 (14.3)	
2—Functions used of the mobile phone/Smartphone:				
Phone calls				4.0 (0.044) *
Yes	69 (98.6)	56 (100)	13 (92.9)	
No	1 (1.4)	0 (0)	1 (7.1)	
Text messaging				1.6 (0.20)
Yes	54 (77.1)	45 (80.4)	9 (64.3)	
No	16 (22.9)	11 (19.6)	5 (35.7)	
Camera				2.1 (0.143)
Yes	42 (60)	36 (64.3)	6 (42.9)	
No	28 (40)	20 (35.7)	8 (57.1)	
Receive videos/photos				3.2 (0.07)
Yes	40 (57.1)	35 (62.5)	5 (35.7)	
No	30 (42.9)	21 (37.5)	9 (64.3)	
Internet search				3.2 (0.07)
Yes	40 (57.1)	35 (62.5)	5 (35.7)	
No	30 (42.9)	21 (37.5)	9 (64.3)	
Applications				3.2 (0.07)
Yes	40 (57.1)	35 (62.5)	5 (35.7)	
No	30 (42.9)	21 (37.5)	9 (64.3)	
Instant messaging				3.2 (0.07)
Yes	40 (57.1)	35 (62.5)	5 (35.7)	
No	30 (42.9)	21 (37.5)	9 (64.3)	
Social networks				2.8 (0.09)
Yes	39 (55.7)	34 (60.7)	5 (35.7)	
No	31 (44.3)	22 (39.3)	9 (64.3)	
Gaming applications				2.8 (0.09)
Yes	39 (55.7)	34 (60.7)	5 (35.7)	
No	31 (44.3)	22 (39.3)	9 (64.3)	
3—Regular access to the Internet				6.8 (0.009) *
Yes (every day)	57 (81.4)	49 (87.5)	8 (57.1)	
No	13 (18.6)	7 (12.5)	6 (42.9)	
4—Frequency of Internet use				9.1 (0.027) *
Every day	52 (74.3)	46 (65.7)	6 (42.9)	
3 or more days/week	2 (2.9)	1 (1.8)	1 (7.1)	
1 day/week	2 (2.9)	1 (1.8)	1 (7.1)	
Less than 1 day/week	14 (20)	8 (14.3)	6 (42.9)	
5—Type of devices used to access Internet				
Personal computer				2.8 (0.08)
Yes	29 (41.4)	26 (46.4)	3 (21.4)	
No	41 (58.6)	30 (53.6)	11 (78.6)	
Tablet				0.02 (0.88)
Yes	16 (22.9)	13 (23.2)	3 (21.4)	
No	54 (77.1)	43 (76.8)	11 (78.6)	
Smartphone				1.2 (0.26)
Yes	53 (75.7)	44 (78.6)	9 (64.3)	
No	17 (24.3)	12 (21.4)	5 (35.7)	
Others				-
Yes	0 (0)	0 (0)	0 (0)	
No	70 (100)	56 (100)	14 (100)	

* Indicates statistically significant differences (*p* < 0.05).

**Table 3:** Preferences of older adults towards technology-based cardiac physical therapy programs.

Variable	Total N (%)	Male N (%)	Female N (%)	χ^2^ (*p*-Value)
1—Interest in receiving continuing advice and support about cardiac rehabilitation through the mobile phone/Smartphone				0.1 (0.71)
Yes	38 (54.3)	31 (55.4)	7 (50)	
No	32 (45.7)	25 (44.6)	7 (50)	
2—Type of Internet communication preferred				
Text messages				0.8 (0.37)
Yes	14 (20)	10 (17.9)	4 (28.6)	
No	56 (80)	46 (82.1)	10 (71.4)	
Video clip				2.1 (0.14)
Yes	11 (15.7)	7 (12.5)	4 (28.6)	
No	59 (84.3)	49 (87.5)	10 (71.4)	
Smartphone App				0.04 (0.83)
Yes	6 (8.6)	5 (8.9)	1 (7.1)	
No	64 (91.4)	51 (91.1)	13 (92.9)	
Internet				1.3 (0.24)
Yes	5 (7.1)	3 (5.4)	2 (14.3)	
No	65 (92.9)	53 (94.6)	12 (85.7)	
Emails				0.16 (0.68)
Yes	18 (25.7)	15 (26.8)	3 (21.4)	
No	52 (74.3)	41 (73.2)	11 (78.6)	
3—Would you like to sign up in a free program that offered these types of communication?				0.92 (0.33)
Yes	38 (54.3)	32 (57.1)	6 (42.9)	
No	32 (45.7)	24 (42.9)	8 (57.1)	
4—How many messages would you like to receive?				5.8 (0.2)
0	32 (45.7)	24 (42.9)	8 (57.1)	
1	12 (21.4)	15 (26.8)	0 (0)	
2	17 (24.3)	13 (23.2)	4 (28.6)	
3	5 (7.1)	3 (5.4)	2 (14.3)	
4	1 (1.4)	1 (1.8)	0 (0)	

**Table 4:** Preferences of older adults about useful advices of technology-based cardiac physical therapy programs.

Variable	Total N (%)	Male N (%)	Female N (%)	χ^2^ (*p*-Value)
Exercise ideas				2.9 (0.4)
Not at all useful	2 (2.9)	1 (1.8)	1 (7.1)	
Little useful	0 (0)	0 (0)	0 (0)	
Somewhat useful	2 (2.9)	1 (1.8)	1 (7.1)	
Quite useful	7 (10)	5 (8.9)	2 (14.3)	
Very useful	59 (84.3)	49 (87.5)	10 (71.4)	
Exercise prompts				14.7 (0.005) *
Not at all useful	3 (4.3)	2 (3.6)	1 (7.1)	
Little useful	6 (8.6)	6 (8.6)	0 (0)	
Somewhat useful	7 (10)	2 (3.6)	5 (35.7)	
Quite useful	3 (4.3)	3 (5.4)	0 (0)	
Very useful	51 (72.9)	43 (76.8)	8 (57.1)	
Exercise programme taught by a virtual therapist				3.4 (0.48)
Not at all useful	6 (8.6)	4 (7.1)	2 (14.3)	
Little useful	8 (11.4)	5 (8.9)	3 (21.4)	
Somewhat useful	6 (8.6)	5 (8.9)	1 (37.1)	
Quite useful	4 (5.7)	4 (7.1)	0 (0)	
Very useful	46 (65.7)	38 (67.9)	8 (57.1)	
Information on local exercise opportunities				4.2 (0.37)
Not at all useful	6 (8.6)	6 (10.7)	0 (0)	
Little useful	2 (2.9)	1 (1.8)	1 (7.1)	
Somewhat useful	8 (11.4)	5 (8.9)	3 (21.4)	
Quite useful	12 (17.1)	10 (17.9)	2 (14.3)	
Very useful	42 (60)	34 (60.7)	8 (57.1)	
Healthy meal ideas and recipes				8.3 (0.015) *
Not at all useful	1 (1.4)	0 (0)	2 (14.3)	
Little useful	0 (0)	0 (0)	0 (0)	
Somewhat useful	8 (11.4)	7 (12.5)	1 (7.1)	
Quite useful	0 (0)	0 (0)	0 (0)	
Very useful	60 (85.7)	49 (87.5)	11 (78.6)	
Practical ideas to manage stress				6.6 (0.036) *
Not at all useful	1 (1.4)	0 (0)	1 (7.1)	
Little useful	0 (0)	0 (0)	0 (0)	
Somewhat useful	0 (0)	2 (3.6)	2 (14.3)	
Quite useful	4 (5.7)	0 (0)	0 (0)	
Very useful	65 (92.9)	54 (96.4)	11 (78.6)	
Setting goals				2.2 (0.68)
Not at all useful	2 (2.9)	2 (3.6)	0 (0)	
Little useful	1 (1.4)	1 (1.8)	0 (0)	
Somewhat useful	7 (10)	5 (8.9)	2 (14.3)	
Quite useful	13 (18.6)	9 (16.1)	4 (28.6)	
Very useful	47 (67.1)	39 (69.6)	8 (57.1)	
Steps to achieve goals				2.0 (0.73)
Not at all useful	5 (7.1)	5 (8.9)	0 (0)	
Little useful	4 (5.7)	4 (5.4)	1 (7.1)	
Somewhat useful	3 (4.3)	2 (3.6)	1 (7.1)	
Quite useful	20 (28.6)	15 (26.8)	5 (35.7)	
Very useful	38 (54.3)	31 (55.4)	7 (50)	
How to overcome cigarette cravings				0.6 (0.95)
Not at all useful	1 (1.4)	1 (1.8)	0 (0)	
Little useful	3 (4.3)	2 (3.6)	1 (7.1)	
Somewhat useful	6 (8.6)	5 (8.9)	1 (7.1)	
Quite useful	16 (22.9)	13 (23.2)	3 (21.4)	
Very useful	44 (62.9)	35 (62.5)	9 (64.3)	
How to remember to take your medications				1.9 (0.74)
Not at all useful	6 (8.6)	6 (10.7)	0 (0)	
Little useful	1 (1.4)	1 (1.8)	0 (0)	
Somewhat useful	14 (20)	11 (19.6)	3 (21.4)	
Quite useful	9 (12.9)	7 (12.5)	2 (14.3)	
Very useful	40 (57.1)	31 (55.4)	9 (64.3)	
Healthy eating tips for takeaways and dining out				4.1 (0.39)
Not at all useful	7 (10)	7 (12.5)	0 (0)	
Little useful	1 (1.4)	1 (1.8)	0 (0)	
Somewhat useful	10 (14.3)	9 (16.1)	1 (7.1)	
Quite useful	12 (17.1)	10 (17.9)	2 (14.3)	
Very useful	40 (57.1)	29 (51.8)	11 (78.6)	
How to link up with others who are living with heart disease				3.7 (0.29)
Not at all useful	46 (65.7)	39 (69.6)	7 (50)	
Little useful	3 (4.3)	3 (5.4)	0 (0)	
Somewhat useful	9 (12.9)	6 (10.7)	3 (21.4)	
Quite useful	0 (0)	0 (0)	0 (0)	
Very useful	12 (17.1)	8 (14.3)	4 (28.6)	

* Indicates statistically significant differences (*p* < 0.05).

## Data Availability

Not applicable.
